# Proteorhodopsin genes in giant viruses

**DOI:** 10.1186/1745-6150-7-34

**Published:** 2012-10-04

**Authors:** Natalya Yutin, Eugene V Koonin

**Affiliations:** 1 National Center for Biotechnology Information, National Library of Medicine, National Institutes of Health, Bethesda, MD 20894, USA

## Abstract

Viruses with large genomes encode numerous proteins that do not directly participate in virus biogenesis but rather modify key functional systems of infected cells. We report that a distinct group of giant viruses infecting unicellular eukaryotes that includes Organic Lake Phycodnaviruses and *Phaeocystis globosa* virus encode predicted proteorhodopsins that have not been previously detected in viruses. Search of metagenomic sequence data shows that putative viral proteorhodopsins are extremely abundant in marine environments. Phylogenetic analysis suggests that giant viruses acquired proteorhodopsins via horizontal gene transfer from proteorhodopsin-encoding protists although the actual donor(s) could not be presently identified. The pattern of conservation of the predicted functionally important amino acid residues suggests that viral proteorhodopsin homologs function as sensory rhodopsins. We hypothesize that viral rhodopsins modulate light-dependent signaling, in particular phototaxis, in infected protists.

This article was reviewed by Igor B. Zhulin and Laksminarayan M. Iyer. For the full reviews, see the Reviewers’ reports section.

## Findings

Many if not all viruses encode proteins that counter-act host defense or more generally affect the functions of cellular systems, presumably tweaking them in a manner that favors virus reproduction. Even viruses with small genomes, for example picornaviruses, typically encode a ‘security protein’ that modifies the host translation system in favor of viral RNA translation [1]. Viruses with larger genomes encode multiple proteins with dedicated functions in the modulation of virus-host interaction at different levels rather than direct roles in virus reproduction [2-5]. A striking example is the presence in numerous cyanophages of genes encoding multiple proteins involved in photosynthesis including complete photosystems I and II [6-8]. These phage-encoded proteins apparently support photosynthesis in infected cyanobacteria and hence promote phage reproduction [9,10]. Here we report the presence in genomes of giant viruses infecting marine unicellular eukaryotes of genes that encode another light-dependent energy-transduction system, proteorhodopsin. We investigate the origin of these genes and discuss their possible roles in the cellular functions of infected protists.

### Bacteriorhodopsins encoded in the genomes of Organic Lake Phycodnaviruses and *Phaeocystsis globosa* virus and their abundant homologs in marine environments

In the course of comprehensive comparative genomic analysis of the giant viruses in the families Mimiviridae and Phycodnaviridae, our attention was caught by 5 viral proteins [one from the nearly complete genome of Organic Lake Phycodnavirus (OLPV) 2, three from fragments of other OLPV genomes [11], and one from the more distantly related *Phaeocystis globosa* virus (PGV)] that showed significant sequence similarity to proteorhodopsins from marine bacteria, in particular the most abundant bacterium in the ocean, *Pelagibacter ubique*. The sequences of these proteins were up to 28% identical to proteorhodopsins (expectation value <e-05); all viral proteins with similarity to proteorhodopsins are currently annotated as ‘hypothetical proteins’ in GenBank although for two of the OLPV proteins the similarity to bacteriorhodopsins is pointed out in a note. Proteorhodopsins represent a distinct, comparatively simple phototrophic system that is of crucial importance in marine ecology [12-14]. These proteins belong to the broader family of bacteriorhodopsins (or Type I rhodopsins) that originally were discovered in Halobacteria (Euryarchaeota) and subsequently identified in diverse bacteria as well as protists and fungi [15]. To our knowledge, proteorhodopsins (or any other rhodopsin superfamily members) have not been previously detected in viruses, so we were interested in a detailed analysis of these sequences.

**Figure 1:**
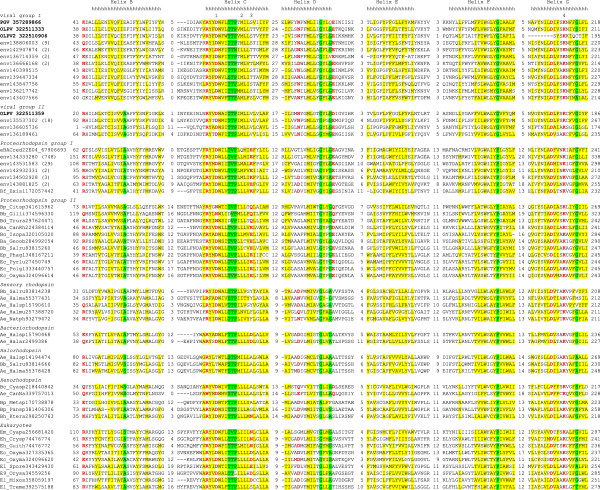
**Conserved sequence blocks in the rhodopsin superfamily.** The conserved blocks are separated by numbers that indicate the length of less well conserved sequence segments which are not shown (see Additional File 1). The alignment columns are colored on the basis of the respective position conservation throughout the superfamily: yellow background indicates hydrophobic residues (ACFILMVWY), red letters indicate polar residues (DEHKNQR), and green background indicates small residues (ACGNPSTV). The transmembrane helices are indicated following transmembrane helix prediction for PGV sequence (helix A is not shown; see Additional File 2 for all 7 predicted transmembrane helices). The functionally important residues are numbered: 1, proton acceptor; 2, position important for spectral tuning; 3, proton donor; 4, retinal-binding amino acid residue. Each sequence is denoted by the corresponding taxon abbreviation followed by the species abbreviation and GenBank Identification (GI) number. Taxa abbreviations: A, Archaea; B, Bacteria; E, Eukaryota; Ae, *Euryarchaeota*; Ba, *Actinobacteria*; Bb, *Bacteroidetes*/*Chlorobi* group; Bc, *Cyanobacteria*; Bd, *Deinococcus*-*Thermus*; Bf, *Firmicutes*; Bh, *Chloroflexi*; Bo, *Planctomycetes*; Bp, *Proteobacteria*; E9, *Viridiplantae*; Ec, *Alveolata*; Eh, *Cryptophyta*; El, *Opisthokonta*; Em, *Glaucocystophyceae*; Ep, *Haptophyceae.* Species abbreviations: are listed in Additional File 3.

Comparison of the putative viral proteorhodopsins to databases of environmental sequences revealed numerous highly similar sequences, some more than 50% identical and with e-values as low as e-64. The much greater similarity between the putative viral proteorhodospins and the environmental sequences, all coming from the Global Ocean Survey (GOS) [16], than between any of these sequences and bacterial proteorhodopsins suggests that the detected environmental sequences are also of viral origin. We collected a representative set of putative viral, bacterial, archaeal and eukaryotic bacteriorhodopsins and constructed a multiple alignment of these sequences (Additional file 1). Inspection of this alignment indicates the conservation of all 7 transmembrane helices that were also independently predicted in the viral proteins (Figure 1 and Additional file 2). Furthermore, the viral sequences contained the invariant lysine residue that is involved in retinal binding as well as the aspartic acid that serves as the proton acceptor; the proton donor glutamate is not conserved; the position known to be important for spectral tuning [17] is occupied by a methionine as it is in some of the proteorhodopsins (Figure 1). The lack of conservation of the proton donor carboxylate indicate that viral proteorhodopsin homologs are sensory rhodopsins rather than light-dependent proton pumps [18,19]. The presence of a hydrophobic residue in the spectral tuning position suggests that viral proteorhodopsins absorb light in the green part of the spectrum [17].

### Origin of the viral proteorhodopsins

**Figure 2:**
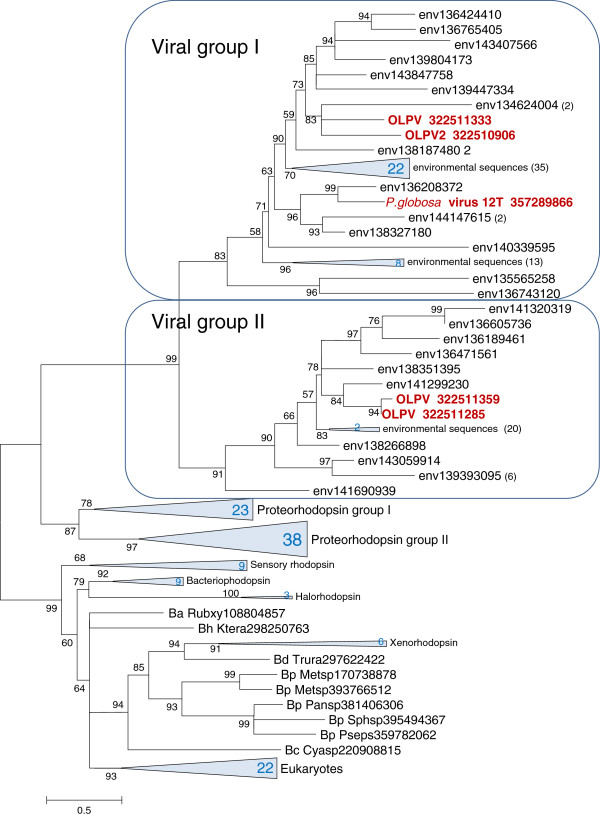
**Phylogenetic tree of the rhodopsin superfamily.** Branches with bootstrap support less than 50 were collapsed. Several large clades are shown by triangles with the number of the collapsed branches shown within the triangle. Numbers in parentheses represent number of environmental sequences clustered into the branch. Each sequence is denoted by the corresponding species abbreviation and GenBank Identification (GI) number. Abbreviations: OLPV, Organic Lake Phycodnavirus; OLPV2, Organic Lake Phycodnavirus 2; env, environmental sequence (marine metagenome); Ba, *Actinobacteria*; Bc, *Cyanobacteria*; Bd, *Deinococcus*-*Thermus*; Bh, *Chloroflexi*; Bp, *Proteobacteria*; Cyasp, *Cyanothece* sp. PCC 7424; Ktera, *Ktedonobacter racemifer* DSM 44963; Metsp, *Methylobacterium* sp. 4–46; Pansp, *Pantoea* sp. Sc1; Pseps, *Pseudomonas psychrotolerans* L19; Rubxy, *Rubrobacter xylanophilus* DSM 9941; Sphsp, *Sphingomonas* sp. PAMC 26617; Trura, *Truepera radiovictrix* DSM 17093. Expanded subtrees for Proteorhodopsin group I and II are shown in Additional file 4.

We used the alignment shown in Figure 1 to construct a phylogeny of bacteriorhodopsins (see Additional file 1 for the full alignment). In the resulting phylogenetic tree, the proteorhodopsin homologs from giant viruses and their environmental homologs form a strongly supported clade with two distinct subclades, each with numerous environmental sequences (Figure 2; see Additional file 3 for details). Taking into account the much higher sequence similarity between the viral sequences and proteorhodopsins as opposed to all other groups of bacteriorhodopsins, the root position can be inferred between two major clades one of which consists of viral and bacterial proteorhodopsins and the other one encompasses the rest of prokaryotic (halorhodopsins, sensory rhodopsins, xenorhodopsins and others) and eukaryotic Group I rhodopsins (Figure 2). This tree structure implies that giant viruses acquired proteorhodospin genes via horizontal transfer from bacteria or more likely from proteorhodospin-encoding eukaryotes.

Notably, *Phaeocystis globosa*, the protist host of PGV, encodes two closely related rhodopsins. However, these rhodopsins confidently group within the eukaryotic branch of Proteorhodopsin Group II (Additional file 4) and accordingly are not the ancestors of the proteorhodopsins of PGV or other viruses. In the tree shown in Figure 2, the viral rhodopsins join the proteorhodopsin clade at its base which at face value seems to suggest ancient acquisition of the proteorhodopsin gene by ancestral giant viruses. However, we cannot rule out that some of the environmental sequences in the ‘viral’ clade actually come from planctonic protists and represent the (still uncharacterized) source(s) of the rhodopsin genes in giant viruses.

### Implications for virus-host interaction in giant viruses

It appears likely that proteorhodopsins of giant viruses modulate phototrophic process in the infected protists. Although proteorhodopsins originally were discovered and characterized in bacteria [13] and subsequently in mesophilic Archaea [20], more recently, members of this family have been identified in several dinoflagellates [21-23]. Notably, in the marine dinoflagellate *Oxyrrhis marina*, proteorhodopsin is the most highly expressed nuclear protein, suggesting a major physiological role(s) [21]. Database searches also indicate the presence of two closely related proteorhodopsins in the prasinophyte *P. globosa* (Figure 1 and Additional file 4). There are no experimental data on the functions of proteorhodopsins in these unicellular eukaryotes. However, by analogy with the well characterized bacterial proteorhodopsins [24], it appears likely that those of the eukaryotic proteorhodopsins that possess the proton donor carboxylate function as light-driven proton pumps involved in ATP synthesis, particularly under oligotrophic conditions, whereas those that lack the proton donor perform sensory functions, in particular in phototaxis [21,25]. By this token, the proteorhodopsins of *P. globosa* are predicted to possess proton-pumping activity (see Figure 1; the second paralogous sequence from *P. globosa* is nearly identical and is not shown).

Viral proteorhodopsins that are predicted to function as sensory rhodopsins could affect signaling and in particular phototaxis in the infected protists, perhaps stimulating relocation of the infected protists to areas that are rich in nutrients required for virus reproduction. Complete sequencing of the genome of *P. globosa* and the still unidentified hosts of OLPV (most likely, also prasinophytes [11]) will show whether the putative viral sensory rhodopsins complement a pre-existing host function or confer a functionality that is new to the host. Given that *P. globosa* is a dominant component of marine phytoplankton and that its population dynamics is substantially affected by viruses [26], viral proteorhodopsin homologs described here, regardless of their exact role(s) that remains to be elucidated experimentally, could be major players in the ocean ecology.

## Conclusions

Proteorhodopsin homologs encoded by giant viruses belong to a distinct proteorhodopsin subfamily that additionally includes numerous uncharacterized sequences from marine environments that are likely to be of virus and/or eukaryotic origin. The viruses probably acquired proteorhodopsin genes from unicellular eukaryotic hosts although the identity of the donors remains unknown. These proteins are predicted to perform light-dependent sensory functions, potentially altering the behavior of the infected protist host, e.g. by inducing phototaxis and perhaps stimulating the host relocation to nutrient-rich areas.

## Methods

Protein sequences were retrieved from the non-redundant database at the National Center for Biotechnology Information (NIH, Bethesda). Reference sequences for halo-, bacterio-, xeno, and sensory rhodopsins were taken from [27]. The non-redundant protein sequence database was searched using the PSI-BLAST program [28], with default parameters and the predicted viral proteorhodopsin sequences used as queries. The reported results reflect searchers performed on 13-15/08/2012. Marine metagenomics blast hits were clustered before the alignment by blastclust ( http://www.ncbi.nlm.nih.gov/Web/Newsltr/Spring04/blastlab.html); a representative (the longest) sequence from each cluster was taken. Protein sequences were aligned using the MUSCLE program with default parameters [29]; columns containing a large fraction of gaps (greater than 30%) and non-homogenous columns defined as described previously [30] were removed from the alignment. The resulting 160-column alignment was used to construct a maximum likelihood phylogenetic tree using the FastTree program with default parameters (JTT evolutionary model, discrete gamma model with 20 rate categories) [31]. Transmembrane helices in proteins were predicted using the TMHMM program [32].

## Competing interests

The authors declare no conflict of interests.

## Authors’ contributions

NY collected the data; NY and EVK analyzed the data; EVK wrote the manuscript which was read and approved by both authors.

## Reviewers’ reports

Reviewer 1: Dr. Igor B. Zhulin, Oakridge National Laboratory and the University of Tennessee

The paper by Yutin and Koonin reports a discovery of proteorhodopsin genes in marine viruses. This is a very interesting finding expanding the repertoire of genes that viruses might carry in order to modify host’s metabolism. As different organisms developed various forms of light-harvesting devices, and at least some of them have been already found in viruses (photosystems I and II), it does not come as a total surprise, and supports the notion that improving host’s conditions promotes phage reproduction. When conditions are right, proteorhodopsin can be a very useful plug-and-play device for energy generation.

The paper is brief, clearly written and goes straight to the point. I do not have any particular comments or concerns rather than it would be usefulto indicate the timing of database searches since NR changes so rapidly and parameters for PSI-BLAST and MUSCLE (presumably, default, but still…) – all in the Methods section

**Authors’ response**: *The details proposed to be included were included.*

Reviewer 2: Dr. Laksminarayan M. Iyer, National Center for Biotechnology Information, NIH

The study details the presence, and analyzes the origins, of proteorhodopsins in certain marine NCLDV viruses. These proteins add to the small, yet interesting, list of laterally acquired genes in large dsDNA viruses that are predicted to alter the response of the infected host to various environmental inputs. The precise biology of how the viral proteorhodopsins contribute to the fitness of the virus and the host should elicit interest among experimental biologists. The analysis can be easily reproduced and the writing is lucid. I only have a minor comment. On the issue of the provenance of environmental sequences most closely related to the viral ones, the authors could consider performing a gene neighborhood analysis, where possible, to see if any predominant associations emerge that might provide clues to the origins of these sequences.

**Authors’ response:***Regrettably, the majority of the environmental sequences are too short for this type of analysis. Those few sequences that were long enough failed to yield useful clues.*

## Supplementary Material

Additional file 1 Secondary structure prediction for viral proteorhodopsin homologs. Click here for file

Additional file 2 **Multiple alignment of bacteriorhodopsins used for the construction of the phylogenetic tree in Figure **2**.** Click here for file

Additional file 3 Supplementary data for Figure 1 and Figure 2. Click here for file

Additional file 4 Expanded phylogenetic trees for Proteorhodopsin groups I and II. Click here for file
