# Supplementary material for: Proteorhodopsin genes in giant viruses
Source: Biol Direct. 2012 Oct 4;7:34. doi: 10.1186/1745-6150-7-34 (PMC3500653; doi:10.1186/1745-6150-7-34)
Supplement: Additional file 3 — Supplementary data for Figure 1 and Figure 2. [file 1745-6150-7-34-S3.doc]

**Species abbreviations for Figure 1**

CanNa, *Candidatus* Nanosalinarum sp. J07AB56; CanRh, *Candidatus* Rhodoluna lacicola; Citsp, *Citromicrobium* sp. JLT1363; Crysp, *Cryptomonas* sp. S2; Cyapa, *Cyanophora paradoxa*; Cyasp, *Cyanothece* sp. PCC 7424; Exisi, *Exiguobacterium sibiricum* 255–15; Geoob, *Geodermatophilus obscurus* DSM 43160; Gilli, *Gillisia limnaea* DSM 15749; Guith, *Guillardia theta*; Halar, *Haloarcula argentinensis*; Halma, *Haloarcula marismortui* ATCC 43049; Halmu, *Halomicrobium mukohataei* DSM 12286; Halsp, *Halobacterium* sp. NRC-1; Isopa, *Isosphaera pallida* ATCC 43644; Ktera, *Ktedonobacter racemifer* DSM 44963; Metsp, *Methylobacterium* sp. 4–46; Mixos, *Mixia osmundae* IAM 14324; Natph, *Natronomonas pharaonis*; Orysa, *Oryza sativa* Indica Group; Oxyma, *Oxyrrhis marina*; Pansp, *Pantoea* sp. Sc1; Phagl, *Phaeocystis globosa*; Polgl, *Polarella glacialis*; Pyrlu, *Pyrocystis lunula*; Salru, *Salinibacter ruber* DSM 13855; Spore, *Sporisorium reilianum* SRZ2; Treme, *Tremella mesenterica* DSM 1558; Trura, *Truepera radiovictrix* DSM 17093; PGAG, Phaeocystis globosa virus 12T; OLPV, Organic Lake Phycodnavirus; OLPV2, Organic Lake Phycodnavirus 2; env, environmental sequence (marine metagenome).

**Phylogenetic tree of the rhodopsin superfamily shown on Figure 2, in newick format:**

(env143011714_3:0.41219,((((env143632167_2:0.36659,(env138754216:0.27877,env134376745:0.33595)0.831:0.07705)0.927:0.11501,env136818837:0.37885)0.764:0.04258,env138806003_9:0.17462)0.925:0.09314,((env136714359_2:0.62406,env138458904:0.48425)0.028:0.05187,env134642457:0.77977)0.045:0.05552)0.873:0.07444,((((env136604100:0.80612,env140400247:0.24284)0.895:0.15981,((((env136208372:0.25302,zn_Phagl357289866:0.31071)0.990:0.24948,(env144147615_2:0.54560,env138327180:0.14937)0.934:0.14663)0.967:0.17472,(env140339595:1.33264,(((env141437690:0.65868,(env139225658:0.41786,(env143426922_2:0.52581,(env143632348:0.25551,env136397672:0.24325)0.926:0.12210)0.954:0.17782)0.499:0.11510)0.669:0.09798,(env136666168_2:0.49041,(env136506649_4:0.34106,env139441433:0.53413)0.914:0.16776)0.670:0.14655)0.962:0.20315,((env135565258:0.99343,env136743120:1.02602)0.964:0.61391,(((((((Bc_Cyasp220908815:1.01785,((((Xeno339757013:0.17439,Xeno339758039:0.19205)1.000:1.27428,(Xeno218440842:0.49251,((Xeno205373523:0.27973,Xeno335427605:0.24773)0.533:0.07804,Bf_Bacco205373524:1.16050)0.762:0.12916)0.801:0.10004)0.913:0.22819,Bd_Trura297622422:0.56846)0.942:0.23747,((Bp_Metsp170738878:0.24776,Bp_Metsp393766512:0.21419)0.997:0.38501,(Bp_Pansp381406306:0.19030,(Bp_Sphsp395494367:0.37327,Bp_Pseps359782062:0.31660)0.065:0.09698)0.998:0.47995)0.934:0.22167)0.850:0.13799)0.944:0.22587,((((((El_Gibze46114444:0.26006,El_Verda346977957:0.20612)0.963:0.22330,(El_Spore343429430:0.36646,El_Treme392579371:0.30585)0.994:0.33410)0.851:0.21626,(E9_Aceac67906884:0.95188,El_Treme392575188:0.66388)0.103:0.11213)0.779:0.04576,(El_Mella328851545:0.43675,(El_Spore323507933:0.57478,El_Mixos358059197:0.29447)0.882:0.11875)0.725:0.11301)0.332:0.07033,((El_Triru327298611:0.44619,El_Penma212526156:0.43006)0.997:0.42454,(El_Fusox342878166:0.47162,(El_Podan171678665:0.21513,(E9_Orysa34559256:0.18704,El_Lepma9858875:0.17296)0.952:0.10931)0.856:0.05368)0.748:0.12957)0.961:0.22942)0.926:0.16400,((Ec_Oxyma324096620:0.53787,Ec_Oxyma327335365:0.32502)1.000:0.56403,((Em_Cyapa268376501:0.75302,Em_Cyapa256681420:0.53959)0.975:0.30435,(Eh_Guith74476770:0.24535,(Eh_Crysp74476774:0.22923,Eh_Guith74476772:0.63992)0.860:0.20067)0.999:0.39641)0.742:0.09706)0.112:0.04617)0.939:0.17976)0.103:0.02946,(Ba_Rubxy108804857:0.35096,Bh_Ktera298250763:0.54743)0.464:0.14827)0.640:0.08306,((Ae_Halwa110666993:0.39782,((Ae_Halsp14194473:0.20375,(Ae_Halut257051402:0.21590,(bacteriorhodopsin_Haloarcula:0.03931,Ae_Halar2499386:0.00920)0.945:0.08739)0.986:0.19350)0.926:0.10402,(Ae_Halch60391838:0.31607,(Ae_uncha155212613:0.33288,(Ae_Halsp15790468:0.13864,Ax_uncar385769539:0.27474)0.978:0.16037)0.054:0.03537)0.956:0.13819)0.743:0.07842)0.920:0.15966,(halorhodopsin__Salinibacter_ruber_83814666:0.34758,(Ae_Halsp14194474:0.26884,halorhodopsin_Haloarcula_marismortui_55378428:0.18125)0.784:0.10895)1.000:1.01441)0.799:0.07874)0.600:0.10320,((sens_III_Haloarcula_marismortui_55377431:0.0,Ae_Halma55377431:0.0):0.93837,((sens_I_Salinibacter_ruber_83814238:0.59934,(sens_I_Halobacterium_salinarum_169236348:0.0,Ae_Halsp15790610:0.0):0.53918)1.000:0.80663,(Ae_Halla222478524:0.68515,((Ae_Halmu257388720:0.17412,(sens_II_Haloarcula_marismortui__55376955:0.00403,Ae_Halhi344211067:0.03668)0.998:0.33360)0.996:0.39976,(Ae_Natgr352126059:0.40762,Ae_Natph93279472:0.30921)0.877:0.15950)0.872:0.14653)0.522:0.15465)0.766:0.09297)0.681:0.18823)0.997:0.67970,((((((xanthorhodopsin_Salinibacter_ruber_83815260:0.00852,Bb_Salru83815260:0.00633)1.000:0.27022,((env140781601_2:0.55660,Bp_SAR32381207017:0.23145)0.927:0.15360,(((Bh_Rossp148657075:0.19040,env143302914:0.67268)0.718:0.08121,Bp_delpr325163228:0.33378)0.815:0.05712,Bd_Thesp384439423:0.32865)0.804:0.05504)0.890:0.07035)0.799:0.08340,(Bo_Isopa320105020:0.29890,(((env141954796:0.23506,Bp_Jansp395764086:0.05044)0.939:0.06826,((((Ec_Polgl333440757:0.10634,(Ec_Oxyma324096614:0.03602,(Ec_Oxyma157093545:0.07782,Ec_Pyrlu27450749:0.12374)0.028:0.01804)0.860:0.04316)0.997:0.21960,(Ep_Phagl348167211:0.03729,Ep_Phagl348167213:0.11094)1.000:0.42429)0.490:0.06883,Bp_Octan254436645:0.32580)0.390:0.05222,((Bp_Marsp126666904:0.30722,Bp_alppr163795412:0.18884)0.592:0.07582,Bp_Sphel383643568:0.21388)0.923:0.06677)0.756:0.02786)0.775:0.06519,((Bx_uncma119713713:0.24001,env138309324:0.27152)0.955:0.17073,env141972440_3:0.58102)0.810:0.08132)0.971:0.13396)0.883:0.09853)0.858:0.03818,((env144191898_5:0.22627,(env136481428_33:0.24524,(env143862209:0.10503,(Ba_actMW224384108:0.12621,Ba_CanRh224384114:0.07887)0.741:0.02151)0.992:0.19131)0.692:0.11508)0.960:0.16296,(Bc_Glovi37519767:0.24406,env139046193:0.21310)0.337:0.05813)0.933:0.11941)0.772:0.09811,(Ba_Geoob284992054:1.15756,(Bp_Citsp341615962:0.73719,((Bo_Phymi383765524:0.43746,Bo_Phymi383767303:0.47234)0.999:0.52268,(Bd_Trura297624471:0.36323,(Bb_Gilli374596330:0.24318,Bd_Trura297625147:0.17417)0.991:0.26821)0.668:0.11573)0.826:0.16918)0.992:0.48421)0.596:0.12623)0.975:0.39944,((((((env143418368_12:0.67632,(((env138469574_2:0.21468,((env136365470_2:0.25452,Ae_uncma77024964:0.18762)0.933:0.09871,(env140018340:0.18811,env141918555:0.28080)0.691:0.11993)0.968:0.14415)0.988:0.19378,(eBACred22E04_67906693:0.20647,Bx_uncAn283574477:0.52980)0.666:0.04882)0.020:0.04037,(Bp_Vibca388601210:0.09079,(env134333280_748:0.06312,Bp_gampr384082807:0.05441)0.864:0.06016)0.945:0.11522)0.226:0.04890)0.987:0.19279,env135916247:0.21460)0.677:0.09116,(env142146276:0.26356,(env142271822_2:0.50141,(Bb_Nitha390443566:0.35532,Bb_Halhy332663718:0.18709)0.685:0.07708)0.263:0.07232)0.947:0.18482)0.494:0.24582,env137418831:1.16720)0.840:0.15594,((env134502928_3:0.54702,(env137575022_2:1.16754,(env143881825_2:0.55137,Bf_Exisi172057442:0.30444)0.589:0.05422)0.852:0.10514)0.891:0.12481,env143531583_29:0.74936)0.623:0.10833)0.762:0.07476,env142932331_2:0.92484)0.787:0.10201)0.871:0.25272)0.998:0.91965,(env141690939:0.55738,((env139393095_6:0.54915,env143059914:0.14390)0.976:0.38108,(env138266898:0.28005,((env135355854_2:0.33618,env135537302_18:0.33243)0.839:0.08595,(((q6_OrgLa322511285:0.00014,q6_OrgLa322511359:0.07396)0.949:0.24933,env141299230:0.25702)0.840:0.14966,(env138351395:0.28449,(env136471561:0.33711,(env136189461:0.38046,(env136605736:0.00012,env141320319:0.26723)0.998:0.41631)0.762:0.14271)0.974:0.19724)0.509:0.06589)0.783:0.03343)0.570:0.12789)0.666:0.14980)0.905:0.32924)0.912:0.30724)0.836:0.32076)0.580:0.23467)0.710:0.03919)0.632:0.03946)0.905:0.07385,(env138187480_2:0.51563,(((env139804173:0.33542,((env136765405:0.29474,env136424410:0.29111)0.949:0.18293,env143407566:0.71053)0.477:0.05876)0.947:0.12412,(env139447334:0.68315,env143847758:0.27566)0.204:0.05687)0.853:0.07340,(env134624004_2:0.99983,(q6_OrgLa322510906:0.46354,q6_OrgLa322511333:0.34968)0.449:0.06560)0.831:0.10803)0.736:0.06120)0.593:0.07094)0.704:0.04424)0.870:0.07312,(((env140824412:0.40683,env140727740:1.18971)0.085:0.11089,(env136587190:1.04502,env138731646:0.26830)0.785:0.18635)0.738:0.05858,((env142947874_2:0.98573,(env141864826:0.53352,env141441420:0.36836)0.107:0.06736)0.352:0.14735,env136217742:0.41626)0.670:0.04796)0.849:0.04700)0.571:0.03717,(env135931882:0.46229,(env136867157:0.72733,env140399515:0.38858)0.128:0.13365)0.410:0.07624)0.794:0.07341);

**environmental (marine metagenome) sequences represented on the Figure 2 tree by their first cluster member:**

134333280 (748) 142154854 134335534 142204569 135075560 135158910 142212768 140056200 144178858 140931952 142919420 135469819 137508079 142969931 143148017 142628359 138533973 142977813 143958068 143831094 134538231 142277482 136359725 140199182 144022183 139620196 142555082 135336501 141210509 136843962 142413122 142659659 135550843 136792206 138227004 139378082 135366914 135368608 136389299 137071457 143400673 135094577 137498264 137545742 142768615 143401356 134409511 134756505 136664439 142013931 142470870 142901448 143019870 143642657 143725956 135398066 135794082 136291883 136900933 137080391 137087196 139595134 142047755 142393900 142408117 142553484 142624827 143269470 143433799 143731020 134889081 134908989 134932817 134972639 135016024 135554529 135667131 135753206 135753756 135914873 135978811 136089828 136371740 136393532 137114542 137712016 137945100 139458870 139803576 140079695 140452658 141505565 141899382 142093008 142231176 142239611 142269989 142409774 142661889 142714620 142862147 142968477 143053897 143110038 143124829 143212368 143442167 143683998 143689121 143816505 143834720 144097146 144203367 144213625 134504681 134974086 135218318 135254931 135450535 135498006 135602039 135762505 135982976 136079302 136083613 136224331 136251679 136285485 136309718 136316043 136329297 136365789 137291856 137959267 141711346 142508855 142557325 142637427 142710238 142732707 142757118 142900996 142950130 143002375 143023946 143077469 143141983 143144550 143790325 143936109 143991087 143991981 134816198 135201634 135341281 135445815 135492000 135534306 135552900 135908157 136301813 136388080 136410555 136624175 136781928 136788532 136804524 138088457 138967107 139805385 141542653 141715225 142412444 142448687 142461121 142471019 142479219 142684016 142708019 142731327 142776989 142873690 142900750 142952990 143030211 143033741 143089965 143277849 143839439 143929001 143934478 143996993 144051788 135824271 136813438 136840758 139514756 140298501 141045391 141145549 142035929 142338016 142403123 142916159 144026610 135849303 136091221 139735116 140961424 134418637 136800557 138379316 139979898 140422278 140742458 143063695 144093866 134480456 134500958 134986643 135641108 135661347 136673867 136803446 141458565 142854328 136122142 137713452 138469333 139386013 143102108 143488442 144112871 135647187 140880391 141834198 143955444 143956388 138136211 139584506 139649858 142036600 142566532 134677573 136896294 137070323 134550278 134796946 134962141 135480256 135773223 136736160 137635118 138043614 138154286 138157422 143266708 143509297 143948941 143962511 135905184 136029823 137147815 138574838 140630377 141234332 141772633 142007514 142845896 143722908 144004260 136858762 138669107 141941310 142600185 142603541 142658875 134636043 137045731 142311593 142551933 143145758 143863294 143940625 134747570 135009844 135367850 135854846 136781829 138068702 139159621 139380391 139537236 143417928 135528317 137814032 140080321 140206133 141387779 142612797 142918947 143150359 143206501 143865403 144148286 134719157 136573015 138094787 141540372 141996634 142585722 143089985 143813279 135670212 136732616 140250971 142346413 134864021 136882738 137603240 139418297 139516707 140091331 142430712 142778202 142947017 143748649 143848315 144208255 135744894 136233422 137131740 139496807 142099703 142107548 142547748 134750278 135233927 135565822 138838293 139496324 139758630 140820353 142628106 142691490 143042999 143447109 134326508 135880352 137897389 139381789 140780213 134786318 135653004 136210646 136739648 138612449 140693147 140975676 142303648 143152209 143217308 134720884 136354906 137154728 138673504 141424312 141852337 141971922 142033876 142076760 142430891 142612335 142827480 142841568 143345072 143518514 143899297 143981963 137006538 139113954 142619530 135896646 137815012 141035714 142022371 138876423 140696059 141202003 141828529 143577001 137903327 139791098 140454654 143135369 143554706 143731782 136018786 136684308 137018897 138413352 138734343 139145917 139823269 139879945 140132808 136490495 137098655 138204105 140796904 141064879 137357203 139145976 141171540 144028795 134352719 135777699 139179797 140170480 142629710 143009796 143154139 144077596 139739343 140849095 143513828 136869816 140788297 140860471 140999498 141458024 143754588 134784934 135426045 136246325 137053218 138575606 142036830 142399814 142520789 142568472 143128158 135900992 136748340 138117232 139680649 140234661 135496130 135885097 136647752 138164274 138302506 138958919 139313621 139371461 139609295 139847272 141403247 134785004 143100682 136404607 136407829 139977094 140295329 141381622 142410311 143838139 144118343 137240935 138127739 139515512 140816505 141693497 142796406 135983504 138790532 139723295 134324414 136027982 138061462 138672818 140785794 142957455 136275233 139645421 137868793 140009078 141834113 134443603 135813318 139483874 140605377 140668143 142601881 142735561 143198938 143449711 143669201 136304843 139035838 139241957 139814275 135638306 135656212 135981998 136684954 140177047 141171597 143550806 143972812 141865109 143029474 134797521 134890202 135738889 134719337 135307530 136808417 139879546 141075435 141531603 137032005 137218087 137550084 138040556 138412849 138496292 139343778 141207880 142602597 142677128 135410696 135805834 141550081 142534220 142691906 142727091 136687982 137394541 138649708 139187161 139259150 139460356 141200256 141612596 142566982 134559228 138704233 139791766 136339249 139917261 138620569 140430278 141926508 141940341 143217012 136828715 139362972 141836481 142441236 142759667 143392183 137786914 139656457 139898697 140083464 140643864 141348538 138137690 139244479 141260912 141272067 135656821 136104474 136385350 135077275 135817003 138100390 134734154 134896992 139517561 140212709 142101984 143135790 134470050 135017474 135780312 136816233 137947983 138113884 140211338 141619847 142482793 142992026 143987702 135024422 138142637 140880257 136400454 138824401 139377910 139911513 143838509 136507175 137612298 138371630 139420059 136857649 136947619 143052854 135354564 137450316 138816010 139860123 139909654 141387780 142024740 137263044 138497764 139412599 143262383 134358393 137931241 139664880 140251414 140399117 140899403 141387127 135793811 137161195 138380033 140011210 135706485 137973095 138497337 139633149 143670908 143721694 139149404 141688999 134640535 141143290 138572898 140532681 140652127 142764652 137332915 138100725 139341880 134943970 135740642 136742667 134517326 139019798 141597047 141716664 142468239 142600011 137101272 138685131 139121253 141673351 142047588 143120536 137521839 139026412 134565894 139380849 141733660 134719233 135588823 135872261 138003160 138138395 139465454 139813581 142458628 136121632 136288866 137351749 135452412 136867667 136953512 137553117 139394259 142258395 137013125 138283945 140822032 141217652 142858713 143871733 140263550 142986719 143229628 140081686 141235580 144054715 135407248 136509054 135552686 136722388 137383048 138062005 142604345 140317944 141415761 143454730 134531516 135559442 136020520 136218086 136666448 136814689 137469601 138866279 139766399 141171855 141537083 142846389 143454541 134595210 136305344 136504940 138047293 137953042 140688396 136944372 140258534 141044448 137704356 141879482 136076015 138003633 135323139 135733062 136916727 139069250 134878022 140710331 141851270 134949564 141361326 136640883 139433528 137075967 136485879

136481428 (33) 136487994 142078884 143377594 136169261 142353427 143308175 138455309 136168940 143891008 143893878 143911209 143890696 136514388 143906406 137757131 136475859 143891584 136568586 136452567 136509176 139468875 142417308 134592215 136155506 136518820 137736726 143341687 134593901 137755326 138454897 136589927

143531583 (29) 142911213 142345754 142307447 135888517 143264993 136746810 142331308 143844834 143953703 140082148 137439744 141844366 136082024 142065881 140483615 134846145 139722097 136099733 137416255 140081879 138083439 141839390 138256554 143505485 136732648 142313000 137547766

135537302 (18) 136844353 136344527 135317452 140025435 135359008 139808262 138332023 144063039 136215672 135786950 139488035 143668244 138202715 136833242 138266897 141805110

143418368 (12) 142848779 143067694 134823254 142723722 143578506 142012298 142654153 138107186 143482504 134438639

138806003 (9) 135788117 141987298 135671267 139736234 143786350 139150994 135896738

135067320 (6) 138555108 139400200 141242590 138555107 141112781

139393095 (6) 141674398 135758914 139790237 136720565 141264781

144191898 (5) 144186547 142067709 134547512 134485821

136506649 (4) 141177895 143359426 139442568

134502928 (3) 134505009 137367286

143011714 (3) 136658821 135813257

141972440 (3) 143371319 140850531

143881825 (2) 138361003

136365470 (2) 135823724

136714359 (2) 139706863

142271822 (2) 143009531

137575022 (2) 140952865

144147615 (2) 135115047

143632167 (2) 137884704

138469574 (2) 139846366

142947874 (2) 140987996

143426922 (2) 134665527

136666168 (2) 139983398

142932331 (2) 134611275

140781601 (2) 142685245

138187480 (2) 135372506

135355854 (2) 135359009

134624004 (2) 139912185
