# Supplementary figures and images for: Proteorhodopsin genes in giant viruses
Source: Biol Direct. 2012 Oct 4;7:34. doi: 10.1186/1745-6150-7-34 (PMC3500653; doi:10.1186/1745-6150-7-34)

## Proteorhodopsin I

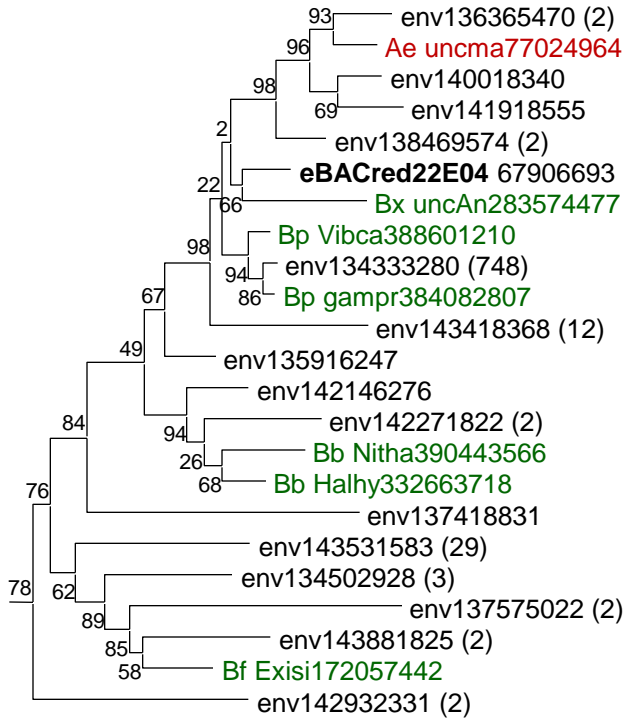

0.5

## Proteorhodopsin II

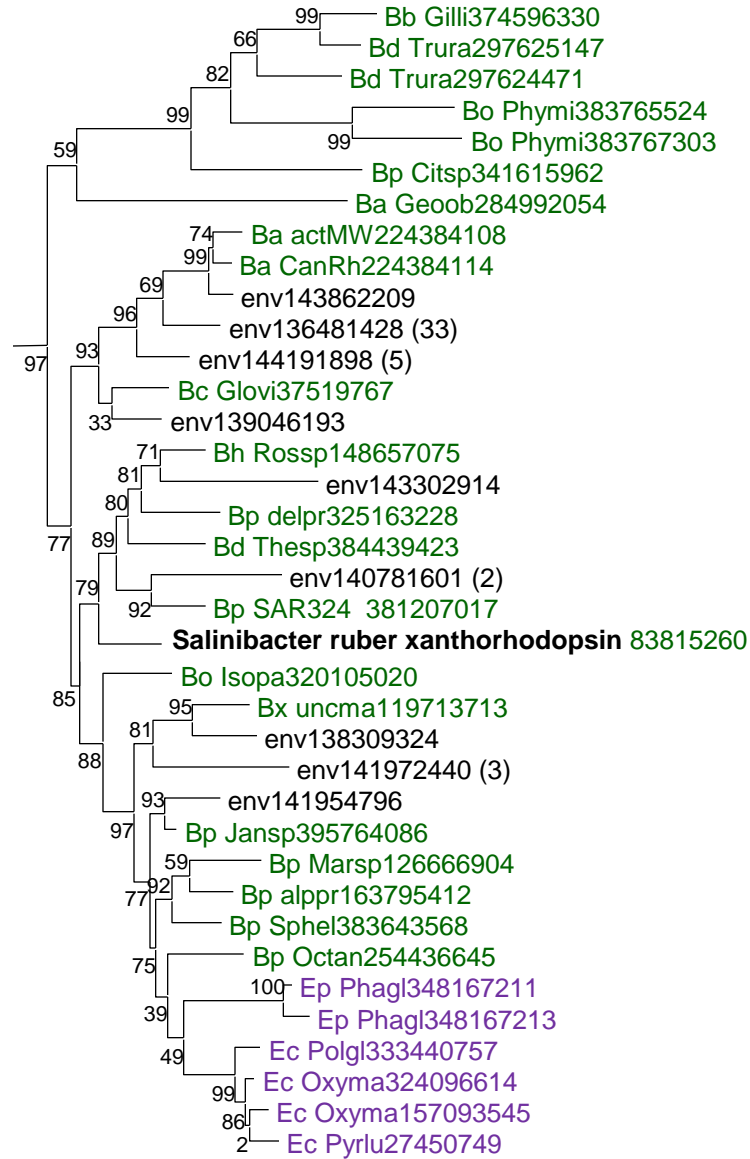

Supplement: Additional file 4 — Expanded phylogenetic trees for Proteorhodopsin groups I and II. [file 1745-6150-7-34-S4.pdf]
