# Supplementary material for: Proteorhodopsin genes in giant viruses
Source: Biol Direct. 2012 Oct 4;7:34. doi: 10.1186/1745-6150-7-34 (PMC3500653; doi:10.1186/1745-6150-7-34)
Supplement: Additional file 2 — Multiple alignment of bacteriorhodopsins used for the construction of the phylogenetic tree in Figure 2. [file 1745-6150-7-34-S2.pdf]

```
                                                                                                        Helix A                             Helix B
   viral group I                 *        20         *        40         *        60         *        80         *       100         *       120         *
PGV 357289866         : -------------------------------------------------------------------MENSFIVKNTMIFSFLIQIIT-LIIGIFAQFIKVPRHKYILKDALLLENIVQFIEAIFYLWFI---- :  62
OLPV 322511333        : ----------------------------------------------------------------------MDNIIMTAYISIFVQIIT-AIISVYGLFIPLNFKDIILREILILELIVQIIEFIFYIWLI---- :  59
env136666168          : ----------------------------------------------------------------------MNFVYFSGILSLVIQVIT-AIIDIYAIKLPIPTNFHLINELVILELIVQIIEASFYVWMV---- :  59
   viral group II
OLPV 322511359        : -----------------------------------------------------------------------------------------MTTGTITFIEALRTKNESVRHILNLETCISVVAAFFYSNFI---- :  41
env135537302          : -----------------MLYTKHNNLLLNLKKIPIMKIIFVKCVQDNFLYILCIMSTKSKVENFSNADNENPYADHYVKMTFYITYVFLLTTGTITFIESMRTSDSKVRHILNLETCISVVAAFFYSKFVSDIE : 117
env136189461          : ---------------------------------------------MGIHVVLDDKLDRCSPLRLLSRMEIEHQSSGLVRLTFYLTYILLLTTGTITLIEALRTTSPAVRHIMNLETCISVVAGYFYSQFI---- :  85
Proteorhodopsin group I
eBACred22E04 67906693 : ----------------------------------------------MRNVLSYALSLLFVAALPSVAFAGDLQANDFVGVTFWIISIS-MVAATLFFFSERSRVSAKWQTSLSVISLVTLIAAVHYFYMR---- :  83
env143531583          : MYFCRKKETPLYICEKNVYRLMSGNKTLRRTALLLTAMAVLAVLVPTALAADAAVDADTGLPAFLSDGAGNDSLELLTFFLFFVGYIS-MGAAFVFFMSERNSVAPEYRTTMTISALIVGIAAFHYYYMR---- : 129
Bf_Exisi172057442     : -------------------------------------------------------------------MEEVNLLVLATQYMFWVGFVG-MAAGTLYFLVERNSLAPEYRSTATVAALVTFVAAIHYYFMK---- :  62
Proteorhodopsin group II
Bd_Trura297624471     : ------------------------------------------------------------MLTMENLLTYSPVSYSIVSNTLTLGYAA-MAAGLVYFVTTSKRAAPPYRLSSTLSAVVMVSAFLALYQLH---- :  69
Bo_Isopa320105020     : --------------------MTTSLLTAGLLLFFAQEGSTAAPAPEAPAAAPAATETSAAPSKEPGLPTLTPAQYSTVYNMFSFTIAS-MGASALFFFLSRNQLAPKYRPAMMVSGLVVSIACYHYFRIF---- : 109
Ep_Phagl348167211     : ---------------------------------------------------------------------MSAGEFDLVYNSLSFTLAS-MMASTLFFWLRLSSVHEKYKTAMTVTGLVTFIAAYHYLRIF---- :  60
                                                                                                            6   6       y

                                                         Helix C                                      Helix D                            Helix E
   viral group I           140         *       160         *       180         *       200         *       220         *       240         *       260
PGV 357289866         : --YFYKEN--VDKIDI--------------------AKYRYYDWFLTTPTMILSVIIYFHYNNSSKKIYYNMITF--FKQDFRKILELWFYNFNMLII-GYLQEIN--IISIL------LSTLIGFYFFGLLF- : 160
OLPV 322511333        : --ITL--Q--SINEDI--------------------TYVRYFDWVLTTPVMLLTTVYFFEYMNSDDGIRKKEIN----DRDYVYLFYICLSNFFMLLI-GYLGETK--QINKM------LTLFGGSFFLFLTF- : 153
env136666168          : --TRF-----NIIKNI--------------------TPFRYYDWAITTPTMLVTLMFYLMFLRDREQGVVSKPFFTEFKTHWRVILKVSILDWLMLLC-GYLGEKH--VFSFL------STTLIGFIPFFLMF- : 156
   viral group II
OLPV 322511359        : --GKLEH---INYEEI--------------------NLNRYVDWAITTPIMLLVLVLAFRVNQTNKA-----------MVKFSDFMIILGMNYGMLGT-GYLGDIG--VIHKT------MGTVLGFLFFG---- : 126
env135537302          : DKEKNNKP--LNYKDI--------------------VVQRYTDWMITTPIMLLVLVLAFAYN--SKT-----------TVNFFTFLVILVFNFGMLLT-GFGGEMGF-IQDLN------LANLGGFIFFFLLY- : 207
env136189461          : --DKINKPGVIDFKDI--------------------NDTRYTDWFITTPLMLLVVLISLSYQN-GKG-----------SVHAKDYLLVVVLNICMLLS-GYMGEKG--IISKP------VGCLVGFLFFIVLF- : 175
Proteorhodopsin group I
eBACred22E04 67906693 : --EVWVET--GESPTV----------------------FRYIDWILTVPLQMIEFYLILA----AVT-----------TVSAGVFNRLLVGTLVMLIG-GYLGEAG--VINAM------LGFIIGMAGWIYIL- : 166
env143531583          : --GVYVDT--QVVSTE----------------------YRYMDWIITVPLMALKFPSLVGKDAITDN-----------KFAGLGFTGICFVGAIIMIGFGYLGEADI-IANET------IALLLGGMGWAMILV : 219
Bf_Exisi172057442     : --DAVGTS--GLLSEI----------------DGFPTEIRYIDWLVTTPLLLVKFPLLLG----LKG-----------RLGRPLLTKLVIADVIMIVG-GYIGESSINIAGGFTQLGLWSY-LIGCFAWIYII- : 158
Proteorhodopsin group II
Bd_Trura297624471     : --QTWLSA--FTFNGEVWEG----------GATAFNNGYRYINWSIDVPILLTQLLIVMG----FTG-----------ARFRRLWLQFVVAGLAMIYT-GYAGQ----FYEATDSARLYLWGAISTAFFLWIL- : 168
Bo_Isopa320105020     : --ESWEAA--YELTGGQYVP----------TGAVFNDAYRYADWLLTVPLLMVELIAVLAL---PKG------------EGRGLLVRLSVAAFFMIAL-GYPGE----IAKYEAGAQYWIFFVLSMIPFLYIL- : 208
Ep_Phagl348167211     : --NSWVDA--YEFPAAAGDGDSIAIHDPVLTGKPFNDAYRYMDWLLTVPLLLIEIIFCCNLSPDETA---------------KKAWALGVSSGLMIIL-GYPGEL---IIEGDMGVRF-MFWFAAMIPFLYVV- : 169
                                                  RY 1W 6t P                                             66   G5 g

                                                    Helix F                              Helix G
   viral group I         *       280         *       300         *       320         *       340         *       360         *       380         *       400
PGV 357289866         : -----YKMFKYYV--VQNKKNYL--------LFFLMFFIWGLYGIAALFNYK----------FKNAFYNILDIFSKNFFGLFLAYLVFTG-------------------------------------------- : 225
OLPV 322511333        : -----YLLYVKYT--KENWMNYI--------VFYFMFLVWFLYGFAFMFPFS----------IKNQMYNILDIVSKNIYSIFIFIVILNQSYKLL--------------------------------------- : 223
env136666168          : -----YLIYKNFA--SQTKTGQT--------IFWYFTAVWAIYGVAAVLPYR----------IKNTIYNILDLFAKNFFGVFLAYVLYKAHQNV---------------------------------------- : 225
   viral group II
OLPV 322511359        : -----GLFYKLNTLRTSNASNDL--------LYGAFFVLWALYGVFYQMEQL----------PRNVGYNVLDLFSKCFVGIYFWAFYAKIFT------------------------------------------ : 195
env135537302          : -----GFIYLKFIYKKYNFDNMI--------IFLSFIILWFFYGVFYPMPYK----------FRNIGYNVLDLFSKCFVGIFFWAYFTKVFTLS---------------------------------------- : 278
env136189461          : -----GFIYFQYVCGSSGLFNYV--------FFGIYVVIWSLYGLVYLLDEE----------HKNMAYNVLDVSAKCFVGLGLWAYFTKIMVL----------------------------------------- : 245
Proteorhodopsin group I
eBACred22E04 67906693 : -----YEIFAGEAGQKSTSCGSVGAQMAFNACRMIVLVGWAIYPLGYLFGYL---MGGVDAASLNLIYNLADFVNKIAFGVFIWAAAIADSAETA--------------------------------------- : 253
env143531583          : ATGLPFGLYDGLGVDNSKIKEEV--KWSTDALRTFILVGWIIYPIGYLFNEN--TYDLGNEEVMGVLYNIADMINKIGFGVVAWMGAKKATAAMA--------------------------------------- : 310
Bf_Exisi172057442     : -----YLLFTNVTKAAENKPAPI--RDALLKMRLFILIGWAIYPIGYAVTLF--APGVEIQLVRELIYNFADLTNKVGFGLIAFFAVKTMSSLSSSKGKTLTS------------------------------- : 252
Proteorhodopsin group II
Bd_Trura297624471     : -----VLVRRTIFDPPDALPERA--AGLMRGVWWVLLGSWLLYPGAYLMPVF--ALSEGGVVARQISFTVADVVSKVIYGVMLSRVAELRSQADGYAHALESETQPNPDRRGGAGVAAVQPQGGRAPRKGRVHR : 293
Bo_Isopa320105020     : -----FVLLTELTKSLERQPENV--RGYISAARLIVLVSWCFYPIAYLASGVGGLSGAQAEVGLQVGYTIADIVAKAVFGVYIYFIARTKSENEGYEVSAQPAVATA--------------------------- : 308
Ep_Phagl348167211     : -----YTLLVGLKEAIASEGNPK-VAAMLTQVCYATVISWCTYPVVYLFPML-GFGGPQAVIAIQLGYCVSDIISKCGVGFLIY-------------------------------------------------- : 246
                                                  W  Y                        5   D   K   g
```
